# Common Signatures of Altered Gene Regulation and Invasiveness of Different Breast Cancer Cell Lines after Matrix Interface Crossing

**DOI:** 10.1002/adhm.202505616

**Published:** 2026-03-05

**Authors:** Cornelia Clemens, Hannah Trampert, Nataliia Kotsiuba, Tilo Pompe

**Affiliations:** ^1^ Institute of Biochemistry Leipzig University Leipzig Germany

**Keywords:** cell migration, extracellular matrix interfaces, invasiveness, phenotype switching, transcriptomic reprogramming, triple‐negative breast cancer

## Abstract

Interfaces between dense tumor tissue and surrounding more porous healthy tissue have been shown to trigger aggressive phenotypes in transmigrating MDA‐MB‐231 breast cancer cells, promoting directional migration, proliferation, and chemoresistance. Here, we show that such interface‐instructed phenotype switching represents a common feature across triple‐negative breast cancer (TNBC) cell lines, highlighting the potential for targeting these matrix interfaces in therapeutic approaches. Using a biomimetic collagen I interface model, we compared the different breast cancer cell lines, namely, MDA‐MB‐231, SUM159PT, and Hs578T, during the transmigration process. The interface‐induced trigger of invasiveness was more pronounced in MDA‐MB‐231 and SUM159PT cells. RNA sequencing revealed shared transcriptional response in all three cell lines, with 228 commonly regulated genes and enrichment of pathways linked to cell cycle, chromatin organization, and DNA repair. Differences in pathway activation reflected the baseline characteristics of the three cell lines. Together, the results demonstrate that the topological and mechanical stimuli of tissue interfaces in general induce transcriptional reprogramming in TNBC cells with features of higher aggressiveness.

## Graphical abstract

Interface transmigration reprograms triple‐negative breast cancer cells, triggering a shared switch toward more aggressive and invasive phenotypes. Using a collagen I interface model, this study identifies shared transcriptional changes involving proliferation, chromatin remodeling, and DNA repair pathways.

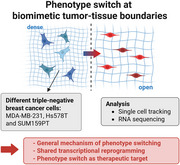

## 1 Introduction

Breast cancer remains the most commonly diagnosed cancer among women worldwide and continues to be a leading cause of cancer‐related mortality, with the global burden expected to increase significantly in the coming decades [1]. Breast cancer is classified into molecular subtypes based on the presence or absence of the hormone receptors for estrogen (ER) and progesterone (PR) as well as the human epidermal growth factor receptor 2 (HER2) [2]. Triple‐negative breast cancer (TNBC) lacks all three receptors. This particularly aggressive subtype is characterized by high heterogeneity, early metastatic spread and limited therapeutic options, as there are still no targeted treatments [3]. Given these challenges and the need for more preclinical studies, investigations on TNBC often rely on well‐established cell line models to reveal mechanisms of disease progression and resistance to therapy. One of the most widely used models is the MDA‐MB‐231 cell line, which plays an important role in studying the migration and invasion behavior of TNBC cells in vitro and in vivo [4, 5]. As this cell line exhibits high motility, invasiveness, and phenotypic plasticity, it serves as a powerful tool to study how TNBC cells respond not only to biochemical signals but also to the physical and mechanical properties of their microenvironment [6, 7, 8]. Besides the widely studied MDA‐MB‐231 breast cancer cell line, other TNBC cell lines, such as Hs578T and SUM159PT are used to capture the heterogeneity of different breast cancers in preclinical studies [8, 9, 10, 11, 12].

The progression of aggressive cancers is closely linked to the ability of tumor cells to invade surrounding tissues and spread to distant sites [13, 14]. This invasive behavior requires cells to overcome a series of mechanical and topological barriers, including migration through different extracellular matrix (ECM) densities and compositions as well as adaptation to dynamic microenvironmental factors [15, 16, 17]. Healthy tissue already exhibits distinct microarchitectural features, such as varying ECM composition, fiber organization, and tissue stiffness, where elastic moduli differ significantly between soft tissues like brain tissue (0.1–1 kPa) and stiffer tissues like pre‐calcified bone tissue (MPa—GPa) [18, 19, 20]. Importantly, as tumors progress, the ECM of the tumor becomes stiffer and more compact due to increased collagen deposition, crosslinking, and fiber alignment, leading to increased differences in topology and stiffness between the tumor tissue and the surrounding healthy tissue [21]. For example, while healthy breast tissue typically exhibits elastic moduli in the range of 0.1–1 kPa, malignant breast tumors can reach 2–20 kPa due to ECM remodeling processes [22].

A critical aspect of cancer cells to invade and adapt to microenvironmental changes is the nucleus, which acts as both a mechanical barrier and a sensor. Nuclear stiffness, largely determined by lamin A levels, scales with tissue stiffness and regulates how effectively cells migrate through confined environments, influencing invasion and cell cycle‐dependent responses [18]. Increased ECM stiffness is also known to enhance contractility, promote mesenchymal migration, and activate mechanotransduction pathways that couple external forces to intracellular signals. These signals include TGF‐β1‐induced myosin IIA‐mediated contractility [23] and stiffness‐dependent focal adhesion kinase signaling, both of which promote increased motility and invasiveness [24]. Furthermore, the transcriptional coactivators YAP and TAZ have emerged as key regulators of mechanotransduction, translating ECM stiffness into changes in gene expression through their nuclear translocation and activation [25].

A substantial difference between the mechanical and topological properties of the tumor tissue and the surrounding healthy tissue not only makes it necessary for cancer cells to adapt to the new microenvironments during invasion, but also leads to the formation of sharp matrix interfaces at the tumor boundary [26, 27]. In recent work, a biomimetic in vitro model was established to replicate these tumor‐tissue interfaces. By using type I collagen matrices with distinct differences in porosity, an interface was created that mimics the abrupt decrease in matrix density with a more open porous topology observed at the tumor margin. Previous studies using the MDA‐MB‐231 breast cancer cell line have shown that such matrix interfaces represent regions of mechanical asymmetry, disrupting nuclear mechanotransduction and promoting a phenotypic switch toward a more aggressive cancer cell behavior [28, 29, 30, 31]. This switch is characterized by increased migration directionality perpendicular to the interface, accompanied by an elongated cell morphology. A persistence of this phenotype switch over several days with reseeding in homogeneous was verified, including demonstration of additional functional phenotypical changes such as elevated chemoresistance toward doxorubicin, increased proliferation, and upregulation of gene clusters associated with increased aggressiveness [29, 30].

While previous studies have extensively characterized the interface‐induced phenotype switching of MDA‐MB‐231 cells at the functional level, including effects on migration directionality, proliferation, and chemoresistance, it remains unclear whether the underlying mechanism is unique to this cell line or represents a common feature of TNBC cells. To address this, the current study compares the three different TNBC cell lines, MDA‐MB‐231, Hs578T, and SUM159PT, to identify shared molecular and migratory signatures induced by interface transmigration. Cell invasion behavior and transcriptomic profiling after matrix interface transmigration were used to assess concordant migratory and transcriptional changes across cell lines, using the switch in migration directionality and cell morphology as phenotypic markers. The results show that crossing matrix interfaces induces a shared switch toward a more directed migration mode in TNBC cells as well as common gene expression patterns. Hence, the study provides a comprehensive picture of the congruent phenotypic responses of TNBC cell lines after migration across a clearly defined ECM interface between a dense and a more porous matrix, similar to a tumor‐tissue boundary.

## 2 Methods

### 2.1 Preparation and Topological Assessment of Collagen I Matrix Interfaces

3D collagen I matrices with well‐defined interfaces that separate compartments of differing porosities were generated on glass coverslips (Ø 20 mm, VWR, Germany) using a previously established protocol [29, 30, 31, 32]. First, the cover slips were modified using 3‐aminopropyltriethoxysilane (Roth, Germany), followed by coating with 0.14% w/w poly(styrene‐*alt*‐maleic anhydride) (PSMA, MW 30 000 g mol^−1^, Sigma–Aldrich, Germany).

Subsequently, two distinct collagen I solutions were prepared by diluting a collagen I stock solution (3.9 mg mL^−1^, Advanced Biomatrix, US) with 0.02 N acetic acid (VWR) and 500 mm phosphate buffer (monosodium and disodium phosphate, pH 7.5, Sigma–Aldrich) on ice. The final collagen I concentrations were adjusted to 1.5 and 3.0 mg mL^−1^, respectively. To create a defined interface between the two matrix compartments, mimicking a tumor‐tissue interface, 50 µL of the first solution was pipetted onto the prepared coverslip and polymerized at 37 °C in a humidified chamber. After washing with phosphate buffer, the second collagen I solution (250 µL) was layered over the first matrix and allowed to polymerize under the same conditions.

To visualize the topology of the collagen matrices, the method outlined by Franke et al. (2014) [33] was used. Collagen fibers were stained with 50 µm 5(6)‐carboxytetramethylrhodamin‐N‐succinimidylester (TAMRA‐SE, VWR) and imaged using confocal laser scanning microscopy (LSM700) equipped with a 40x immersion objective (both, Carl Zeiss Microscopy, Germany). Image Stacks of 50 µm thickness were captured at 5 µm z‐intervals (resolution: 1024 × 1024 px, 0.16 µm px^−1^). A custom MATLAB‐based image analysis tool was used to quantify the mean pore and fibril diameters.

### 2.2 3D Cell Culture

Three breast cancer cell lines, MDA‐MB‐231, Hs578T (both from DSMZ, Germany), and SUM159PT (Cytion, Germany), were cultured according to the suppliers’ guidelines. All cell lines were maintained in DMEM supplemented with 10% FCS (Merck, Germany) and 1% Zellshield (Minerva Biolabs, Germany). For Hs578T cells, the medium was additionally enriched with 0.01 mg mL^−1^ human insulin. Cell cultivation was performed in a humidified incubation unit under standard conditions (37 °C, 5% CO_2_).

### 2.3 Live Cell Tracking of Breast Cancer Cells in Collagen I Interface Matrices

To analyze migration across biomimetic tumor‐tissue interfaces, MDA‐MB‐231, Hs578T, and SUM159PT cells were embedded within the denser compartment of collagen I interface matrices. Prior to embedding, cells were harvested, counted, and resuspended in 500 mm phosphate buffer (pH 7.5). Next, the cell suspension was combined with the collagen I solution and 0.02 N acetic acid, ensuring a final cell density of 1 × 10^5^ cells per interface matrix. The resulting cell‐collagen mixture was layered on top of the previously polymerized, cell‐free collagen I compartment as described earlier. Polymerization was carried out in a humidified chamber. Subsequently, the matrices were washed with pre‐warmed 1× PBS.

Following matrix assembly, the collagen I matrix interfaces were transferred to 12‐well plates and overlaid with DMEM (supplemented as described in Section 2.2 for each cell line). The matrices were pre‐incubated for 24 h under standard cell culture conditions (37 °C, 5% CO_2_, 95% humidity). For cell migration analysis across the collagen interfaces, the well plates were placed in an inverted fluorescence microscope (Axio Observer Z1, Carl Zeiss Microscopy) equipped with an incubation chamber to maintain stable environmental conditions.

Time‐lapse imaging was performed over a 7‐day period. Image stacks covering a total depth of 250 µm were acquired at 5 µm z‐intervals every 10 min in transmission mode using a 10x dry objective at the matrix interfaces. Each captured image had a resolution of 692 × 520 px with a pixel size of 1.253 µm.

Manual single‐cell tracking was conducted using the MTrackJ plugin within ImageJ [34, 35]. Cell trajectories were subsequently used for analyzing migration directionality and speed. Only cells that could be continuously tracked and that crossed the collagen matrix interface were included in the analysis. For these cells, trajectories were segmented into two sub‐tracks: from the start of the track to the interface and from the interface to the end of the track. Directionality was quantified for each sub‐track as the ratio of net displacement to total track length. A directionality of 1 indicates highly directional migration, while a directionality of 0 indicates random migration behavior.

### 2.4 Gene Expression Analysis using RNA Sequencing

To obtain a sufficient number of cells for RNA sequencing, breast cancer cell populations before and after interface crossing needed to be expanded, as was also performed in a previous study [30]. After 7 days of cultivation within interface matrices under standard cell culture conditions, the matrix compartments were separated using tweezers. Cells from each compartment were then isolated by enzymatic digestion with Collagenase IV (200 U mL^−1^, Life Technologies, US) in HBSS buffer with Ca^2+^ and Mg^2+^ (VWR). The resulting cell suspensions were subsequently seeded into the previously prepared T25 cell culture flask coated with 2.0 mg mL^−1^ collagen I for further expansion for 7 days. Each condition (transmigration vs. non‐transmigration) was performed with two independent biological replicates.

Following expansion, RNA was extracted from the breast cancer cell populations using phenol/chloroform extraction. Therefore, RNA Tri‐Liquid (BioSell, Germany) was directly added to the collagen matrices containing the expanded cell populations, followed by a 5‐min treatment in an ultrasound bath. Subsequently, chloroform was added, and the aqueous phase was collected for RNA Isolation using the ReliaPrep RNA Clean‐Up and Concentration System (Promega, US), following the manufacturer's protocol. RNA quality was assessed using a Fragment Analyzer 5300 (Agilent Technologies, US), followed by RNA sequencing on an Illumina NovaSeq 6000 (Illumina, US). Both procedures were conducted at the Core Unit DNA Technology at the Medical Faculty of Leipzig University. RNA samples with a low RNA quality number were excluded from further analysis.

RNA sequencing data were analyzed using R (v4.5.0) using *Bioconductor* packages. Raw gene counts were normalized and variance‐stabilized using *DESeq2* [36], and low‐quality samples and low‐count genes were excluded. Potential batch effects were assessed via exploratory principal component analysis (PCA). Given the absence of detectable batch effects and the focus on pathway‐level analyses, no further batch correction was applied. Differentially expressed genes (DEGs) were identified with an adjusted *p*‐value (FDR) ≤0.05 and |log_2_ fold change| ≥1. Venn diagrams of DEGs were generated using the *VennDiagram* package [37]. Pathway enrichment analysis was performed using Reactome Analysis (*ReactomePA* package) [38] with input genes converted to Entrez IDs using *org.Hs.eg.db* [38]. Significant pathways were identified and summarized by hierarchical parent–child relationships using Reactome's pathway structure. Average log_2_ fold changes were calculated for each pathway.

### 2.5 Third‐Party Libraries used Under R

**Table:** 

Library	Version	Source
clusterProfiler	4.16.0	[39]
DESeq2	1.48.1	[36]
GGally	2.2.1	[40]
org.Hs.eg.db	3.21	[41]
pheatmap	1.0.12	[42]
ReactomePA	1.52.0	[38]
tidyverse	2.0.0	[43]
VennDiagram	1.7.3	[37]

### 2.6 Statistical Analysis

All experiments were performed as triplicates unless otherwise stated. Statistical significance was analyzed using the Kruskal–Wallis test in GraphPad Prism 9 (GraphPad Software, Inc., US) or R (v4.5.0). For pairwise comparisons following a significant Kruskal–Wallis result, Dunn's post‐hoc test was performed. Statistical levels are set at: *p* <0.05 (^*^: *p* <0.05; ^**^: *p* <0.01; ^***^: *p* <0.001).

## 3 Results

### 3.1 Recapitulation of the Influence of Biomimetic Collagen I‐Based Tumor‐Tissue Interfaces on Breast Cancer Cell Behavior

Understanding the interplay between metastasizing cancer cells and their surrounding microenvironment is essential for developing new treatment options for aggressive cancer types such as TNBC. For successful metastasis, cancer cells must cross various physical barriers, such as blood vessel walls and basement membranes. Notably, the first interface between the dense, stiff tumor tissue and the softer, more porous surrounding healthy tissue represents a critical regulator of invasive behavior [28, 29, 30, 31]. To better understand the process of cancer cell migration across clearly defined ECM interfaces, we previously established a 3D collagen I‐based model that mimics such tumor‐tissue interfaces in vitro [29].

**FIGURE 1:**
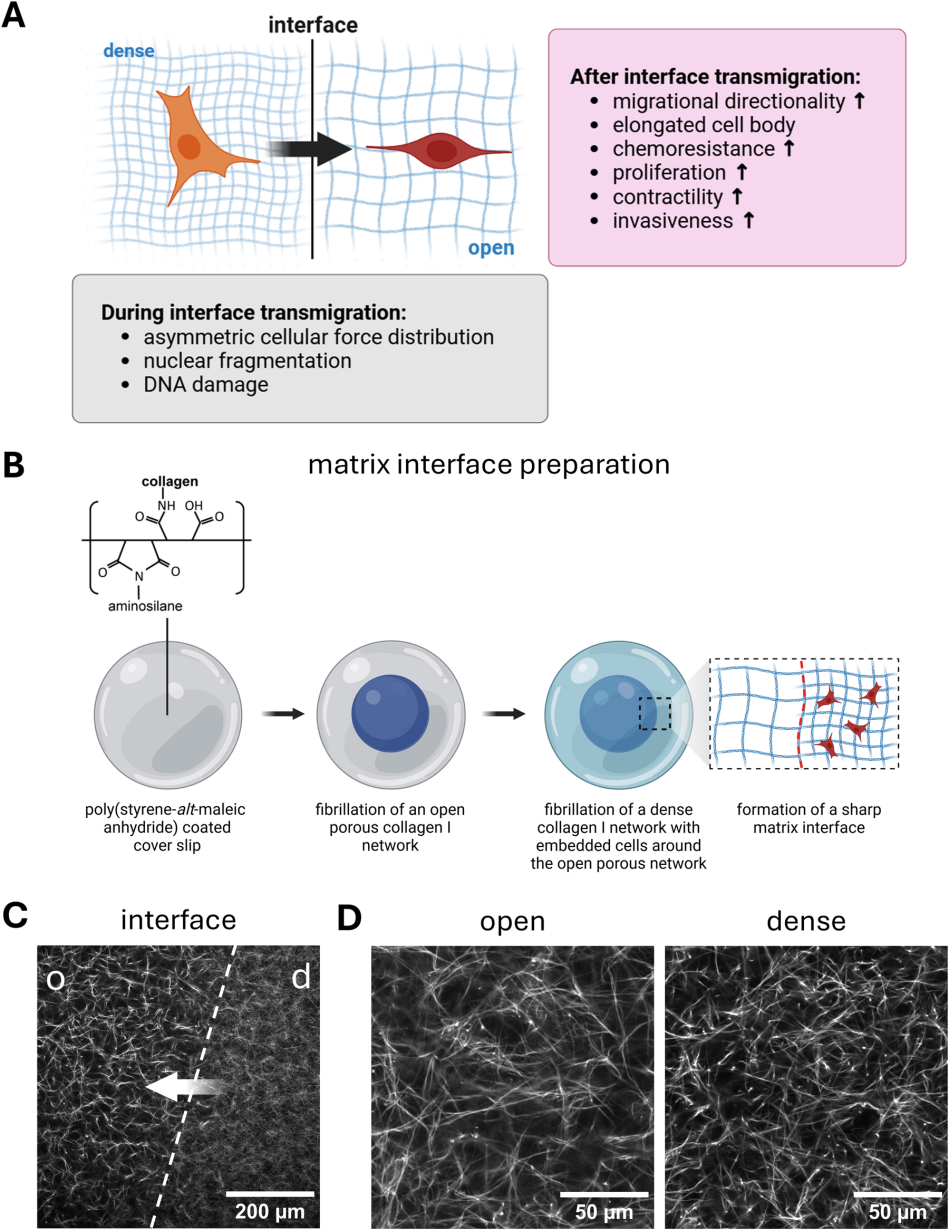
Collagen I‐based biomimetic tumor‐tissue interfaces induce phenotype switching in MDA‐MB‐231 breast cancer cells. (A) Summary of previous results found for MDA‐MB‐231 cancer cells during and after matrix interface transmigration. (B) Schematic illustration of the preparation of d→o (dense to open) collagen I matrix interfaces on poly(styrene‐alt‐maleic anhydride)‐coated coverslips. A sequential fibrillation strategy using two collagen I solutions with different concentrations was applied to form first a dense matrix and subsequently an open porous matrix around the dense matrix, generating a sharp interface. (C) A representative confocal laser scanning microscopy image of TAMRA‐SE‐stained collagen I matrix interfaces. The dense compartment is labeled with ‘d’ and the more porous compartment with ‘o’. The dashed line indicates the sharp interface. A white arrow indicates the direction of cell migration. (D) Representative confocal laser scanning microscopy images of the open and dense matrices next to the interface with higher magnification.

This system consists of two adjacent collagen I compartments with defined differences in fibril density, creating a sharp interface that recapitulates the structural transition from dense tumor ECM to the more open porous ECM of healthy tissue. Using this model, we and others have shown that when invasive MDA‐MB‐231 breast cancer cells transmigrate from the dense into the more porous compartment, they undergo changes in phenotype (Figure 1A) [28, 29, 30, 31]. This transmigration included enhanced migration directionality perpendicular to the interface as well as an elongated cell body [29]. Furthermore, transmigrated MDA‐MB‐231 cells showed an elevated proliferation rate and increased resistance to doxorubicin, highlighting the clinical relevance and importance of understanding this switch in cellular behavior. Transcriptomic analyses revealed an increase in differentially expressed genes associated with a more aggressive phenotype [30]. Importantly, crosslinking experiments that produced interfacial matrices with equal stiffness but differing porosity in both compartments demonstrated that not the difference in stiffness between the dense and more open porous compartments is the critical regulator, but the steep increase in pore size at the defined matrix interface [29]. All these phenotype changes were shown to be persistent during reseeding and cultivating the transmigrated cells in different matrices over several days.

Recent investigations have further elucidated the underlying molecular mechanisms driving this interface‐induced phenotype switching. When breast cancer cells cross sharp collagen I matrix interfaces with differing pore sizes, they generate strong, localized forces that deform the matrix and mechanically challenge the nucleus. This leads to DNA damage and disrupted nuclear mechanotransduction, including misregulated Emerin and YAP signaling, which is correlated with a more contractile and aggressive phenotype [30, 31]. Single‐cell tracking and clonal proliferation studies combined with modeling further showed that these interfaces act instructively on all cells, rather than filtering pre‐existing subpopulations [32].

To test the generality of this phenotype switching at matrix interfaces for TNBC and to gain deeper mechanistic insight into how matrix interfaces instruct the switch toward metastatic phenotypes, we now compared three breast cancer cell lines, namely MDA‐MB‐231, SUM159PT, and Hs578T during transmigration of the collagen I matrix interfaces (Figure 1B). The interface matrices featured the same topology as published earlier, with a pore diameter of 5 µm in the open porous compartment and 4 µm in the dense compartment (Figure 1C,D; Figure S1) [31]. In the following, the matrix densities dense and open are abbreviated as ‘d’ and ‘o’. Matrix interface crossing is indicated as ‘d→o’ for ‘dense to open’.

### 3.2 Characterization of Morphology and Invasive Behavior of Three Breast Cancer Cell Lines

The cell lines MDA‐MB‐231, SUM159PT, and Hs578T all originate from TNBC tumors but differ notably in their histological classification as well as migratory behavior [8, 44, 45, 46]. These differences make them particularly suitable for analyzing subtype‐specific signaling pathways underlying invasion and the response to microenvironmental cues.

The MDA‐MB‐231 cell line was derived from a pleural effusion of metastatic adenocarcinoma [44] and is one of the most widely used TNBC models. It shows fast individual cell migration, high proliferation, and strong cellular contractility [47, 48]. This cell line is highly invasive in spheroids within collagen I matrices and is known for its pronounced tumorigenicity and metastatic capacity in vivo [8, 47]. The SUM159PT cell line originates from a primary anaplastic carcinoma [45] and exhibits fast individual cell migration and high proliferation, too [48]. It is highly invasive in spheroid and collagen I matrix models but displays only moderate tumorigenicity and low metastatic potential in vivo [8]. Hs578T cell line, derived from a primary carcinosarcoma of the breast [46], shows only slow individual migration and intermediate proliferation [47, 48]. Compared to the other two cell lines, it is less invasive in spheroids in collagen I as well as poorly tumorigenic and metastatic in vivo [8, 47]. However, Hs578T cells exhibit high cellular contractility, similar to MDA‐MB‐231 cells [47, 49]. Consistent with these functional differences, analysis of a published transcriptomic dataset [50] reveals that all three cell lines display a broadly mesenchymal gene expression profile with downregulation of MET markers, while Hs578T and SUM159PT show higher expression of several EMT‐associated genes and collagen‐binding integrins compared to MDA‐MB‐231 (Figure S2). Thus, the cell lines share a common mesenchymal TNBC phenotype but differ in the magnitude and composition of EMT‐ and adhesion‐related programs.

**FIGURE 2:**
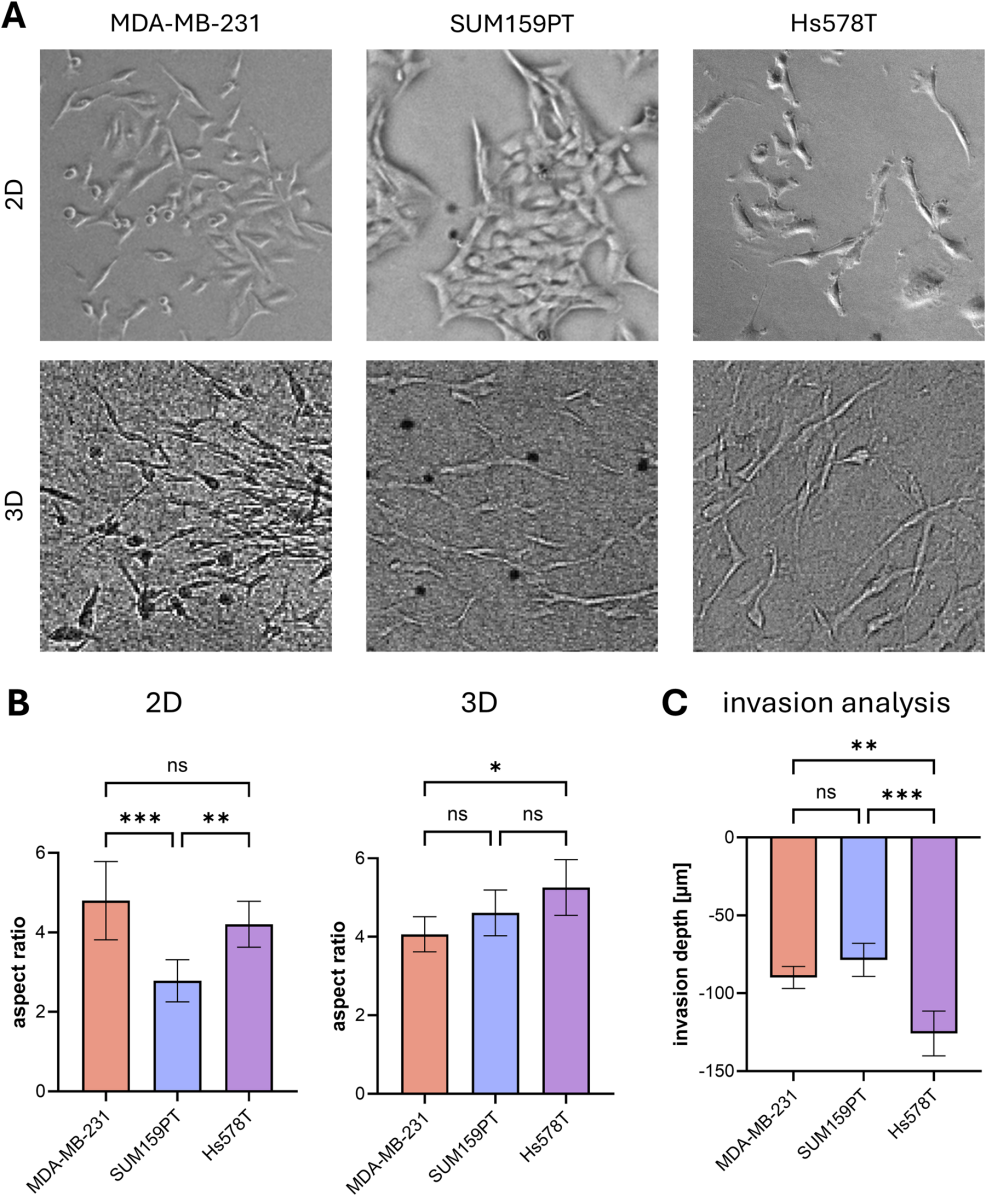
Characterization of breast cancer cell lines MDA‐MB‐231, SUM159PT, and Hs578T. (A) Phase contrast microscopy images of MDA‐MB‐231, SUM159PT, and Hs578T cultivated either in cell culture flasks or in 2.5 mg mL^−1^ collagen I matrices. Image size: 250 µm × 250 µm. (B) Morphology analysis of the three cell lines in 2D cell culture flasks and in 2.5 mg mL^−1^ collagen I matrices. 30 cells per cell line were quantified. (C) Invasion assay of the three cell lines into 2.0 mg mL^−1^ collagen I matrices. After seeding the cells on top of the collagen matrices and incubating for seven days, the nuclei were stained using DAPI, and the invasion depth was quantified using fluorescence microscopy.

Representative phase contrast microscopy images show that in standard 2D cell culture flasks, MDA‐MB‐231 and Hs578T cells exhibit a spindle‐shaped morphology and grow predominantly as single cells (Figure 2A). SUM159PT cells also display an elongated shape, but, in contrast, tend to form cell clusters. When embedded in 3D collagen I matrices, all three cell lines maintained an elongated morphology and were predominantly found as single cells, including SUM159PT. Quantification of cell morphology confirmed these observations. In 2D, the aspect ratio showed no significant difference between MDA‐MB‐231 and Hs578T, both of which maintained an elongated shape. In contrast, SUM159PT cells appeared significantly rounder due to their clustering, despite an overall elongated phenotype (Figure 2B). After embedding and cultivation in 3D collagen I, the aspect ratio was nearly comparable across all cell lines, with Hs578T cells exhibiting a slightly stronger elongation of the cell body relative to the other two cell lines, consistent with previous reports [47].

Taken together, these findings confirm that all three TNBC cell lines share an overall elongated, invasive‐like morphology, yet display subtle differences in shape and organization that may influence their invasive capacity. Notably, the shift of SUM159PT cells from forming clusters in 2D to adopting a single‐cell, elongated shape in 3D demonstrated the importance of physiologically relevant biomimetic 3D environments.

To compare the invasiveness of the breast cancer cell lines and their ability to migrate in collagen I matrices, a standard invasion assay [51] was performed. Therefore, the breast cancer cells were seeded on top of collagen I matrices, and after seven days of incubation, nuclear positions were quantified by fluorescence staining with DAPI. The invasion assay confirmed that all three cell lines are capable of invading collagen I matrices (Figure S3A). MDA‐MB‐231 and SUM159PT showed comparable invasion depths, consistent with their shared morphological properties in 3D. In contrast, Hs578T cells invaded significantly deeper into the collagen I matrices than the other two cell lines. This increased invasion depth aligns with the stronger elongation observed in 3D and is consistent with previous studies that showed an increased cellular contractility and ECM remodeling for Hs578T cells using β1/α2 integrin‐mediated traction forces [47]. Additionally, recent work has demonstrated that Hs578T cells display a relatively high 3D invasion speed and persistence in collagen matrices, yet these in vitro behaviors contrast with their comparatively lower tumorigenicity in vivo [8].

In summary, these results highlight the invasive potential of all three TNBC cell lines in collagen I matrices, providing a robust basis for further investigations of their migration and invasion across complex mechanical and topological features, such as defined matrix interfaces.

### 3.3 Adaptation of Migration Directionality and Cell Morphology During Matrix Interface Crossing

**FIGURE 3:**
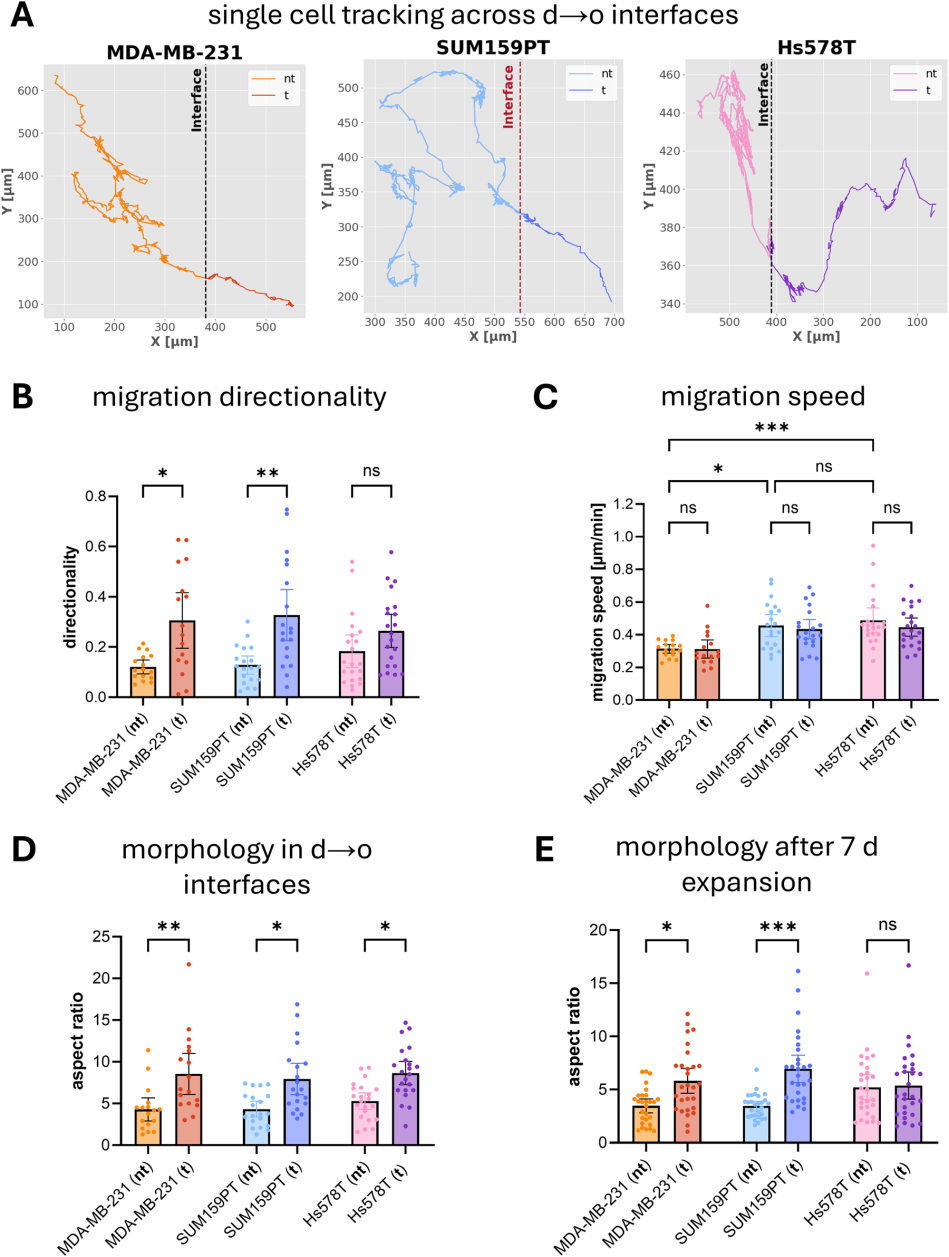
Live cell tracking of breast cancer cell lines crossing sharp collagen I matrix interfaces. (A) Representative migration trajectories of single MDA‐MB‐231, SUM159PT, and Hs578T cells migrating across an interface between a dense (left) and a porous (right) collagen I matrix. (B) Quantification of migration directionality before and after transmigration across the interface (N = 20). (C) Migration speed calculated from single‐cell trajectories (N = 20). (D) Aspect ratio of cell bodies within the matrices next to the interface after 7 days of culture, measured before and after crossing the interface (N = 20). (E) Aspect ratio of cells reseeded and expanded for 7 days in homogeneous 2.0 mg mL^−1^ collagen I matrices to assess persistence of morphological changes (N = 30). nt – non‐transmigrated, t – transmigrated.

In previous work, we demonstrated that as MDA‐MB‐231 cells migrate across collagen I matrix interfaces from a dense to an open porous matrix, they undergo phenotype switching. This involved a change in migration behavior, characterized by a more directed migration away from the interface as well as a switch in morphology into a more elongated shape oriented perpendicular to the interface [30]. To investigate whether similar effects occur for all three TNBC cell lines, we performed single‐cell tracking experiments (Figure 3A). Based on the extensive functional characterization of interface‐induced phenotype switching in MDAMB231 cells reported previously [28, 29, 30, 31, 32], we use changes in migration directionality and cell morphology here as phenotypic markers to identify analogous interface‐induced phenotypic changes in SUM159PT and Hs578T cells.

Quantification of migration directionality confirmed previous findings for MDA‐MB‐231 cells with increased directionality after interface crossing. Similarly, a significant increase in directionality was observed for the SUM159PT cell line (Figure 3B). Hs578T cells showed only a slight tendency of directionality increase, without being significant. Besides the changes in directionality, migration speed remained constant during interface crossing for all three cell lines (Figure 3C). Furthermore, absolute migration speed was in the same range for all cell lines, with SUM159PT and Hs578T cells displaying slightly higher average speed than MDA‐MB‐231 cells (MDA: 0.31 ± 0.08 µm min^−1^, SUM: 0.47 ± 0.14 µm min^−1^, Hs: 0.45 ± 0.13 µm min^−1^). We quantified the morphology of the breast cancer cells within the matrices before and after transmigration across the matrix interface, too (Figure 3D). All three cell lines exhibited a significant increase in cell body elongation, indicating that the biomimetic tumor‐tissue interface induces a common morphological adaptation. To assess whether this elongation is transient for a few hours and depends on the specific matrix or is a persistent characteristic of the transmigrated cells, cells were isolated from both matrix compartments (before and after transmigration), reseeded into a homogeneous collagen I matrix, and cultured for another seven days (Figure S3B). Aspect ratio remained elevated for transmigrated MDA‐MB‐231 and SUM159PT cells (Figure 3E), suggesting a persistent morphological change, whereas the Hs578T cell line reverted to its original morphology.

In summary, the migratory and morphology analyses demonstrated that the defined interface between the dense and open porous matrices exerts a lasting influence on MDA‐MB‐231 and SUM159PT cells, inducing both directional migration and cell body elongation. The persistence of the elongated morphology over several days in homogeneous matrices after reseeding suggests that these cells may undergo stable epigenetic reprogramming, which could prime them for long‐term invasiveness. In contrast, Hs578T cells show only a transient morphological response, with only a weak, non‐significant effect on migration directionality. This indicates that while all three lines sense and react to the topological interface, the magnitude and persistence of this adaptation depend on cell line‐specific traits. For example, Hs578T shows weak tumorigenicity in vivo and lower proliferation compared to MDA‐MB‐231 and SUM159PT, suggesting a distinct baseline phenotype that may shape the degree of phenotypic response to interface crossing, as will be further supported by differences in baseline gene expression levels discussed in Chapter 3.4. These differences may help to uncover the molecular pathways that determine how tumor cells integrate topological cues into persistent invasive behavior.

### 3.4 RNA Sequencing Reveals Common but Distinct Transcriptional Responses to Matrix Interface Transmigration across TNBC Cell Lines

To unravel a broader picture of possible common pathway regulation changes in the three cell lines associated with transmigration across collagen I matrix interfaces, we performed RNA sequencing on MDA‐MB‐231, SUM159PT, and Hs578T cells after 7 days of expansion post‐transmigration (Figure S3B). Previous studies on MDA‐MB‐231 cells have shown that expansion of transmigrated cells in homogeneous matrices over 7 days maintained their changes in phenotype and gene expression [30]. Differential gene expression analysis was conducted using DESeq2, applying thresholds of an adjusted *p*‐value < 0.05 and |log_2_ fold change| >1.

**FIGURE 4:**
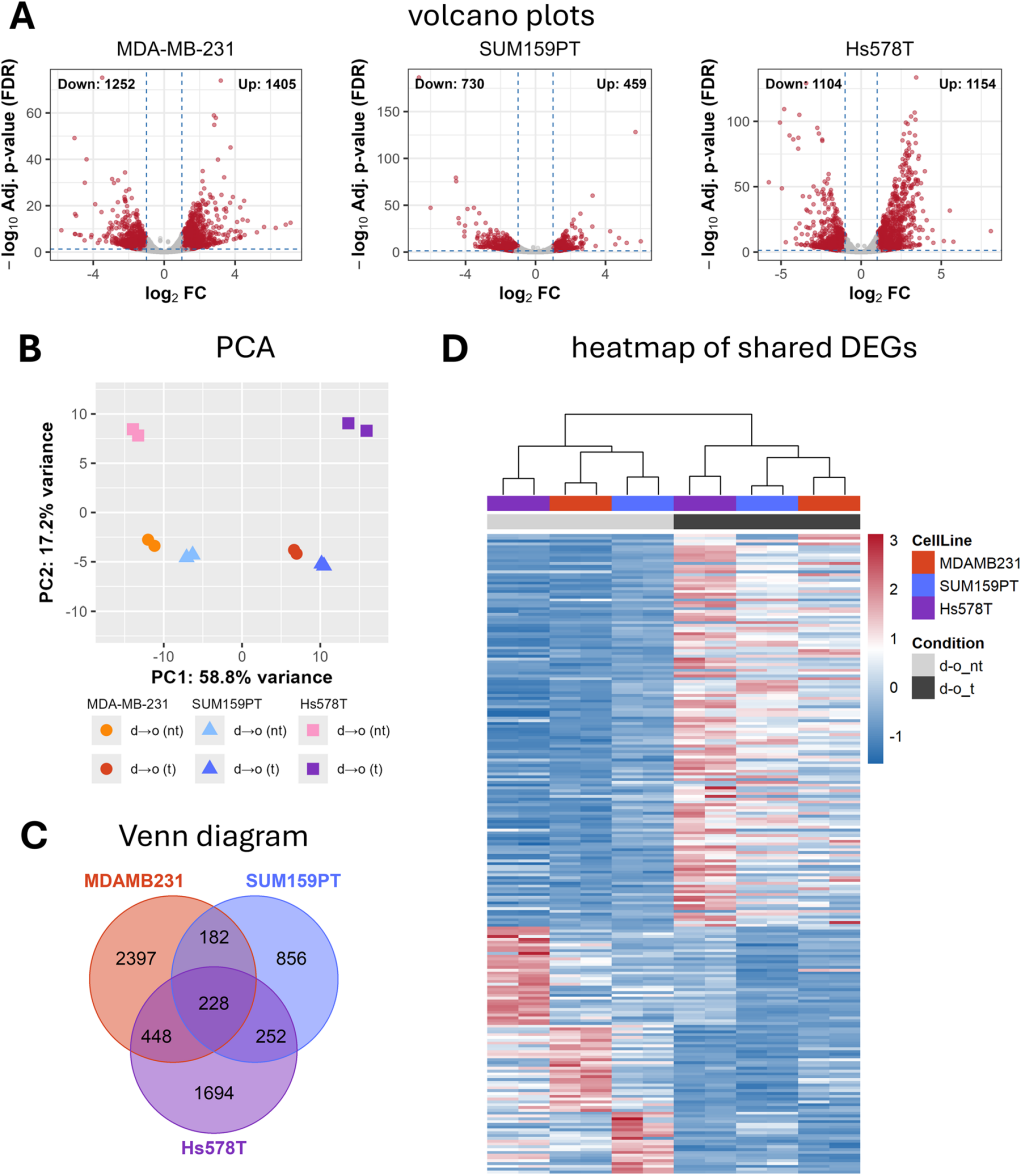
RNA sequencing of three TNBC cell lines before and after transmigration across the matrix interface. (A) Volcano plots showing differentially expressed genes (DEGs) for MDA‐MB‐231, SUM159PT, and Hs578T cells after transmigration across a d→o matrix interface compared to non‐transmigrated cells (thresholds: *p* <0.05, |log_2_FC| >1). (B) Principal component analysis (PCA) showing the overall transcriptional separation between non‐transmigrated (d→o(nt)) and transmigrated (d→o(t)) populations for each cell line. (C) Venn diagram depicting the overlap of DEGs among the three cell lines. (D) Heatmap of the shared DEGs. nt – non‐transmigrated, t – transmigrated.

Volcano plots comparing gene expression before and after transmigration across the d→o interface demonstrate substantial transcriptional changes for all three cell lines (Figure 4A). MDA‐MB‐231 and Hs578T cells exhibit a larger number of differentially expressed genes compared to SUM159PT, indicating a more pronounced transcriptional response to the matrix interface (MDA‐MB‐231: 1405 upregulated and 1252 downregulated; Hs578T: 1154 upregulated and 1104 downregulated; SUM159PT: 459 upregulated and 730 downregulated). In contrast, comparison of cells cultured in homogeneous dense and open collagen I matrices yields way less gene expression changes across all cell lines, suggesting that matrix topology alone has a limited impact on transcriptional profiles (Figure S4A).

A subsequent principal component analysis (PCA) further supported these findings (Figure 4B). Cells that transmigrated across the interface formed distinct clusters separate from the non‐transmigrated counterparts in each cell line. Notably, MDA‐MB‐231 and SUM159PT clustered closely, suggesting comparable transcriptional responses, whereas Hs578T formed a distinct cluster, highlighting its unique transcriptional adaptation to the interface, which aligns with its different adaptation behavior in terms of morphology and migration directionality (Figure 3). In contrast, cells cultured in homogeneous dense and open collagen matrices clustered by cell line but not by matrix density, confirming that the matrix topology per se has little to no effect on gene expression in this setting (Figure S4B).

Venn diagram analysis of differentially expressed genes (DEGs) following transmigration revealed substantial transcriptional remodeling in all three cell lines, with 3255 DEGs in MDA‐MB‐231, 1518 in SUM159PT, and 2622 in Hs578T. Despite these cell line‐specific responses, 228 genes were commonly regulated across all three cell lines after transmigration (Figure 4C). In contrast, comparison of cells cultured in homogeneous dense versus open matrices resulted in markedly fewer DEGs for each cell line and no shared DEGs across all three models (Figure S4C). These results suggest that matrix interface crossing triggers similar gene expression changes across the three cell lines, indicating a common biological response rather than cell line‐specific or random effects. Heatmap visualization of these shared genes revealed clear baseline differences between cell lines before transmigration (Figure 4D). However, post‐transmigration cells exhibited a more congruent gene expression profile, with Hs578T cells showing a stronger upregulation. This elevated transcriptional response in Hs578T contrasts with its weaker phenotypic changes observed in migration directionality and morphology (Figure 3B,D,E).

**FIGURE 5:**
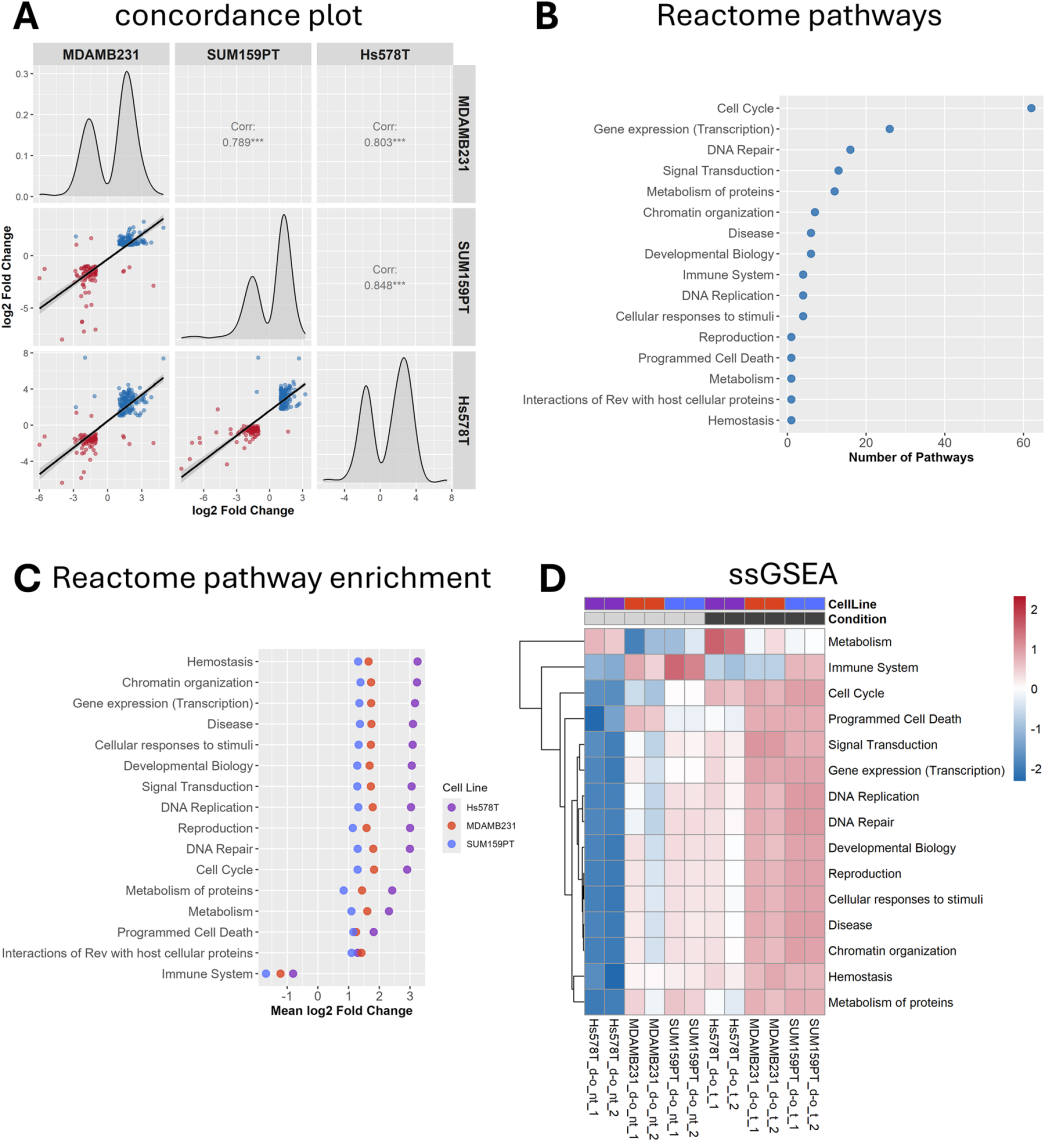
Pathway analysis of the 228 shared genes between the three cell lines. (A) Concordance plot showing pairwise log2 fold changes of the shared differentially expressed genes across MDA‐MB‐231, SUM159PT, and Hs578T cell lines after transmigration. (B) Pathway classification of the shared genes based on Reactome analysis, showing the number of sub‐pathways grouped under each level 1 ancestor category. (C) Scatter plot of Reactome pathway enrichment results for each cell line, displaying log2 fold changes for enriched level 1 ancestor pathways. (D) Heatmap of single‐sample gene set enrichment analysis (ssGSEA) scores for the shared pathways across non‐transmigrated (d→o (nt)) and transmigrated (d→o (t)) conditions. ssGSEA scores were z‐score normalized per pathway, with higher values indicating relatively increased pathway activity following transmigration. nt – non‐transmigrated, t – transmigrated.

To further characterize the shared transcriptional changes induced by transmigration across the biomimetic tumor‐tissue interface, we examined the correlation changes among the 228 commonly regulated genes. Concordance plots revealed strong positive correlations between each pair of cell lines, indicating that these genes are similarly regulated across the three lines (Figure 5A).

Reactome pathway analysis of this shared gene set highlighted a predominant enrichment in pathways related to the cell cycle (Figure 5B). This includes processes like mitotic phases, cell cycle checkpoints, DNA synthesis, and chromosome maintenance (Figure S5), reflecting the activation of proliferation and the tight control of the cell cycle following transmigration. This finding aligns well with previous observations in MDA‐MB‐231 cells, where an increase in proliferation as well as enrichment in gene networks related to cell division was reported after matrix interface crossing [30].

Additionally, pathways involved in gene expression and transcription were enriched, encompassing epigenetic regulation, RNA polymerase activity, and histone modification complexes (Figure 5B). This suggests extensive transcriptional reprogramming and chromatin remodeling in response to matrix interface crossing. Enrichment of DNA repair pathways, such as base excision repair and homology‐directed repair, further indicates engagement of genomic maintenance mechanisms, potentially reflecting cellular stress or DNA damage that emerged during migration through the d→o matrix interface. This is consistent with earlier findings that showed an increased DNA damage and nuclear deformations during matrix interface crossing in MDA‐MB‐231 cells [30]. Key signal transduction pathways were also identified, including WNT, Rho GTPases, Notch, and nuclear receptors, all of which are known to regulate cytoskeletal dynamics, cell fate decisions, and migration [52, 53, 54, 55]. Comparing the overall magnitude of regulation across these highest‐level Reactome pathways, Hs578T cells exhibited consistently higher mean log2 fold changes relative to MDA‐MB‐231 and SUM159PT (Figure 5C). This enhanced transcriptional response complements the heatmap data (Figure 4D) and underscores that although Hs578T displays a subtle migratory phenotype switch, it exhibits a pronounced molecular reaction to matrix interface crossing, similar to the other two TNBC cell lines.

Further supporting these findings, single‐sample gene set enrichment analysis (ssGSEA) was performed to evaluate pathway activation at the individual sample level. The ssGSEA results demonstrated that pathways related to the previously found enriched Reactome pathways are consistently upregulated in all three cell lines following transmigration (Figure 5D). Notably, Hs578T cells, which showed lower baseline pathway activity prior to transmigration, exhibited a pronounced increase in enrichment scores after interface crossing (Figure S6). In contrast, MDA‐MB‐231 and SUM159PT cells displayed higher baseline pathway activity and a more moderate increase post‐transmigration. This observation suggests that Hs578T cells maintain a more quiescent or less proliferative transcriptional state under baseline conditions, consistent with their characterization as a comparatively weakly aggressive TNBC. Following matrix interface crossing, however, Hs578T cells display a marked induction of these pathways, indicating the possibility of also stimulating this cell line to a strong molecular response. Despite this pronounced transcriptional activation, the phenotypic outcome remains limited, at least for the investigated features of cell elongation and migration directionality over the short time of 7 days (Figure 3B,D,E). This pattern suggests that although Hs578T cells show a pronounced increase in pathway activation after transmigration from a low baseline, their overall activity still remains below the level required to elicit a substantial change in migratory behavior (Figure S6). In contrast, MDA‐MB‐231 and SUM159PT cells, with higher baseline activity and greater absolute pathway activation, displayed more pronounced functional changes.

To assess the clinical relevance of this transmigration‐associated transcriptional program, we next analyzed its expression in patient tumors from the published METABRIC TNBC cohort [56] (Figure S7A–F). Signature scores derived from the 228 shared DEGs were highest in basal‐like tumors and increased with tumor grade, consistent with elevated proliferative activity in more aggressive disease. Other clinical variables, including tumor size, age at diagnosis, lymph node involvement, and Nottingham Prognostic Index, showed only minimal or modest associations. Notably, although the signature was derived from Claudin‐low TNBC cell lines (MDA‐MB‐231, SUM159PT, Hs578T), its expression pattern in patient tumors aligned more closely with high‐grade, basal‐like disease. These findings indicate that the transmigration‐associated transcriptional program captures a proliferation‐dominated state characteristic of aggressive TNBC and may serve as a molecular indicator of tumor aggressiveness.

To further capture cell line‐specific responses beyond the shared gene set, an additional Reactome pathway enrichment was performed using all differentially expressed genes for each cell line individually (3255 DEGs in MDA‐MB‐231, 1518 in SUM159PT, and 2622 in Hs578T) (Figure S8A–C). Overall pathway patterns were comparable to those observed for the shared genes. However, Hs578T and MDA‐MB‐231 cells exhibited stronger enrichment of cell cycle and DNA repair pathways than SUM159PT, with Hs578T showing the most pronounced transcriptional upregulation. Notably, Hs578T cells displayed broad enrichment across multiple DNA repair mechanisms, whereas MDA‐MB‐231 cells showed a more restricted DNA repair response, and SUM159PT cells exhibited minimal enrichment. These differences suggest cell line‐specific engagement of DNA repair pathways following interface transmigration, with Hs578T cells mounting a particularly comprehensive genomic maintenance response.

Closer inspection of the cell‐line specific DEGs revealed only modest changes in genes related to EMT or collagen binding (Figure S9). Minor changes were observed in EMT markers, with MDA‐MB‐231 showing CDH1 upregulation and CDH2 downregulation. Further examination of representative genes from the 228 shared gene set showed consistent upregulation of cell cycle regulators (*CCNB1*, *CCNA2*, *CDC20*, *PLK1*, *MCM4*, *MCM5*, *MYBL2*) and DNA repair genes (*FANCD2*, *RAD51AP1*, *BLM*), highlighting the proliferation‐dominated nature of the shared transcriptional program and the engagement of genomic maintenance mechanisms. In addition to the proliferation‐dominated response, we observed upregulation of genes strongly linked to invasion and aggressive phenotypes, including *CCN1* [57], *CCN2* [58], *MMP11* [59], *CEMIP* [60], *EPAS1* [61], *PTN* [62], *CTNNAL1* [63], *COL6A2* [64] and *CXCL8* [65], highlighting that the transcriptional program also engages pathways associated with ECM remodeling, cell migration and metastatic potential. Hallmark gene set [66] analysis further supported a robust activation of cell cycle programs, while coverage of EMT [66] and metastasis‐associated genes [67] remained limited (15%–20% of genes per cell line for EMT, <15% for metastasis‐associated genes) (Figure S10).

It is important to note that RNA sequencing was performed on cells that had been expanded for 7 days following isolation from the biomimetic interface matrix. Thus, the observed transcriptional programs likely reflect persistent and stable phenotypic adaptations rather than acute mechanical stress responses to the topological asymmetry of the interface. The enrichment in pathways related to cell cycle progression, chromatin remodeling, and DNA repair suggests that interface crossing may induce long‐lasting reprogramming toward increased proliferation, survival, and transcriptional adaptability.

## 4 Discussion & Conclusion

This study builds upon previous work demonstrating that interfaces between dense tumor tissue and softer, more open porous healthy tissue serve as triggers for (instructive) phenotype switching in transmigrating MDA‐MB‐231 breast cancer cells. Earlier findings revealed that after crossing a matrix interface from dense to open matrices, the breast cancer cells adopt a more invasive phenotype characterized by elevated proliferation, increased chemoresistance, and stronger migration directionality perpendicular to the interface. This phenotype switch was accompanied by an elongated morphology, a feature commonly associated with increased invasiveness.

In our new study, phenotype switching in MDA‐MB‐231 cells was investigated alongside other triple‐negative breast cancer (TNBC) cell lines with differing histopathological backgrounds and invasive capacities to determine whether phenotype switching after interface transmigration is unique to MDA‐MB‐231 cells or whether it represents a common response of breast cancer cells to matrix interface crossing. To this end, we compared three TNBC cell lines: MDA‐MB‐231, exhibiting a highly invasive character and mesenchymal‐like phenotype; SUM159PT, known for its high proliferative and invasive potential with both single‐cell and collective invasion modes; and Hs578T, characterized by slower proliferation and lower invasiveness in vivo.

Our migration studies revealed that both MDA‐MB‐231 and SUM159PT cells exhibit a significant switch to more directed migration after crossing the collagen I matrix interface, without altering their migration speed. Morphologically, these cells exhibited an elongated cell shape, being persistent also in re‐seeded matrices up to 7 days after interface transmigration, indicating a stable phenotypic adaptation. In contrast, Hs578T cells responded only weakly and non‐significantly in terms of migration directionality and exhibited only transient cell elongation after interface crossing. These observations suggest that cell line‐specific variability exists in the capacity to translate interface‐induced stimuli into persistent, invasive behavior. Moreover, it supports our previous conclusion that differences in matrix elasticity and correlated cell contractility play a minor role, while the observed phenotype switching is primarily driven by the topological cues of the matrix interface [29]. The baseline differences and similarities among MDA‐MB‐231, SUM159PT, and Hs578T cells, representing a broadly mesenchymal profile (Figure S2), as well as the high cellular contractility of MDA‐MB‐231 and Hs578T cells and low contractility of SUM159PT cells, combined with a shared significant switch of MDA‐MB‐231 and SUM159PT cells is fully in line with this conclusion.

RNA sequencing of cells expanded for seven days after transmigration uncovered a significant transcriptional response across all three cell lines. Differential expression analysis identified 228 genes regulated after interface crossing in all three cell lines, highlighting a shared gene expression program. Reactome pathway enrichment underscored the involvement of cell cycle pathways, confirming an interface‐associated switch toward higher proliferative activity across all cell lines. Pathway enrichments encompassed metabolism, hemostasis, programmed cell death, and immune system processes, reflecting a broad phenotypic remodeling.

Notably, pathways involved in chromatin organization and epigenetic regulation of gene expression were significantly enriched, suggesting that the dense‐to‐open interface induces long‐term chromatin remodeling and transcriptional reprogramming. The activation of DNA repair pathways, such as base excision and homology‐directed repair, points toward engagement of genomic maintenance mechanisms, possibly in response to mechanical stress and DNA damage experienced during transmigration. This is consistent with prior reports showing increased DNA damage and nuclear deformation in MDA‐MB‐231 cells migrating through dense‐to‐open interfaces [30]. We hypothesize that the mechanical stress experienced during interface transmigration induces DNA damage in specific chromatin regions, transiently altering 3D genome architecture. The surrounding histone landscape critically influences the recruitment of DNA repair complexes and determines which repair pathways are employed [68, 69]. Importantly, TNBC cell lines differ in their DNA repair capacities, as previously reported [70, 71, 72]. The repair process itself may incorporate new histones, reshaping chromatin structure over multiple cell cycles. Inheritance of such alterations in chromatin structure and function may lead to “chromatin fatigue” in subsequent cell lineages, potentially contributing to long‐lasting transcriptional reprogramming and phenotypic adaptation following interface transmigration [68]. Differences in DNA repair pathway engagement and buffering capacity could therefore underlie the observed variations in phenotypic responses in our study. In line with this, Hs578T cells have been reported to exhibit distinct activation of DNA damage response and homologous recombination‐associated pathways compared with other TNBC models [70, 71, 72], which may facilitate efficient recovery from transmigration‐induced stress and result in more transient phenotypic changes, whereas MDA‐MB‐231 and SUM159PT cells may experience more persistent alterations.

Hs578T cells also demonstrated higher mean log2 fold changes in most enriched pathways relative to MDA‐MB‐231 and SUM159PT, despite their weaker phenotypic response. Single‐sample gene set enrichment analysis (ssGSEA) revealed that Hs578T cells exhibited substantially lower basal expression of these pathways prior to interface crossing. Although their post‐transmigration induction of these pathways is comparatively stronger, the absolute activity levels only reach those seen in MDA‐MB‐231 and SUM159PT cells before transmigration. This aligns well with the existing literature, which describes Hs578T as a slower‐proliferating, less invasive TNBC line [8, 73, 74]. Furthermore, the relatively low activity levels of Hs578T after transmigration, which are close to the baseline observed in MDA‐MB‐231 and SUM159PT before transmigration, correspond with our observation that Hs578T cells exhibit a weaker and more transient response in migration directionality and cell elongation compared to MDA‐MB‐231 and SUM159PT cells. This likely allows Hs578T cells to repair DNA damage efficiently, restoring cellular homeostasis and limiting persistent phenotypic changes. In contrast, MDA‐MB‐231 and SUM159PT cells, with higher baseline activity but comparatively limited DNA repair capacity, may experience cumulative DNA damage, potentially promoting longer‐lasting transcriptional and phenotypic alterations.

Taken together, our results demonstrate that the sharp decrease in fibrillar collagen density of the ECM at the tumor‐stroma interface exerts a common impact on migrating breast cancer cells, inducing phenotypic and transcriptional changes across multiple TNBC lines. However, the degree to which these molecular changes translate into functional adaptations varies between cell lines, likely reflecting intrinsic differences in baseline signaling and DNA repair competence. Our study confirms that transmigration across interfaces between matrices with different fibrillar collagen densities, in the direction from dense to more porous matrices, triggers phenotypic and transcriptional reprogramming in breast cancer cells, being at least persistent over several days and cell divisions. Increases in invasiveness and transcriptome profiles associated with higher aggressiveness appear to be a common regulatory response in breast cancer cells migrating into surrounding healthy tissue. The shared response across all cell lines highlights a biomedically relevant effect, emphasizing the important role of ECM topological changes in cancer cell progression and their consequences.

Future investigations should focus on capturing the molecular dynamics occurring precisely at the moment of interface crossing, which may uncover key regulatory mechanisms triggered by the sharp decrease in porosity, including nuclear deformation, DNA damage, and chromatin remodeling. Investigating additional cell types, like healthy cells, may clarify whether interface‐induced phenotype switching requires underlying genomic instability [75, 76] or a distinct mechanical character of the nucleus [77, 78], relating it to a feature restricted to cancer cells or being more broadly applicable.

## Author Contributions

C.C. contributed to the conceptualization of the study and was involved in the investigation and formal analysis. She also led the writing of the original draft and participated in editing the manuscript. H.T. contributed to the investigation and performed formal analysis. N.K. was involved in the investigation and carried out formal analysis. T.P. contributed to the conceptualization of the study and took part in writing the original draft, as well as reviewing and editing the manuscript.

## Conflicts of Interest

The authors declare no conflicts of interest.

## Supporting information




**Supporting File**: adhm71005‐sup‐0001‐SuppMat.pdf.

## Data Availability

All data and home‐built MATLAB scripts reported in this work are available from the corresponding author upon reasonable request. The MATLAB script for network topology analysis is freely available at https://git.sc.uni‐leipzig.de/pe695hoje/topology‐analysis.
