# Supplementary material for: Common Signatures of Altered Gene Regulation and Invasiveness of Different Breast Cancer Cell Lines after Matrix Interface Crossing
Source: Adv Healthc Mater. 2026 Mar 5;15(18):e05616. doi: 10.1002/adhm.202505616 (PMC13176507; doi:10.1002/adhm.202505616)

Common Signatures of Altered Gene Regulation and Invasiveness of Different Breast Cancer Cell Lines after Matrix Interface Crossing

Cornelia Clemens[a], Hannah Trampert[a], Nataliia Kotsiuba[a], Tilo Pompe[a]*

Supplementary data

Supplementary Figure 1

Supplementary Figure 1: Topology analysis of collagen I-based interface matrices. Collagen matrices were stained with TAMRA-SE and imaged by confocal laser scanning microscopy (cLSM). Pore sizes were significantly larger at 1.5 mg/mL compared to 3.0 mg/mL ($p < 0.001$), while fibril diameters showed no significant difference ($p = 0.056$). Within the d→o interfaces, pore sizes were significantly increased in the transmigrated vs. non-transmigrated compartment ($p < 0.001$), whereas fibril diameters remained unchanged ($p = 0.443$). Bold letters indicate the compartment (dense - d or open - o) which was analyzed. The data shown are reused from C. Clemens et al., Biomater. Sci. (2025).

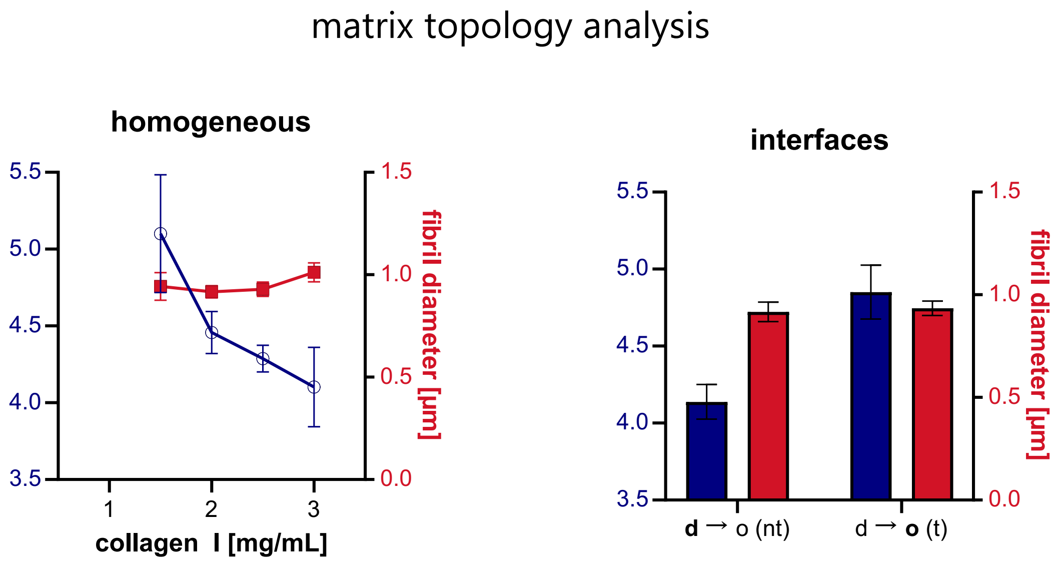

Supplementary Figure 2

Standardized expression of selected integrin subunits and EMT/MET markers in MDA-MB-231, SUM159PT and Hs578T cells derived from a published transcriptomic dataset (Heiser, LM et al. (2012) *Subtype and pathway specific responses to anticancer compounds in breast cancer*. Proc. Natl. Acad. Sci. U. S. A. 109:2724-9). Heiser et al. profiled baseline gene expression across a large panel of ~50 breast cancer cell lines using microarray-based transcriptomics prior to drug treatment. Expression values were z-score-standardized per gene to enable comparison across cell lines.

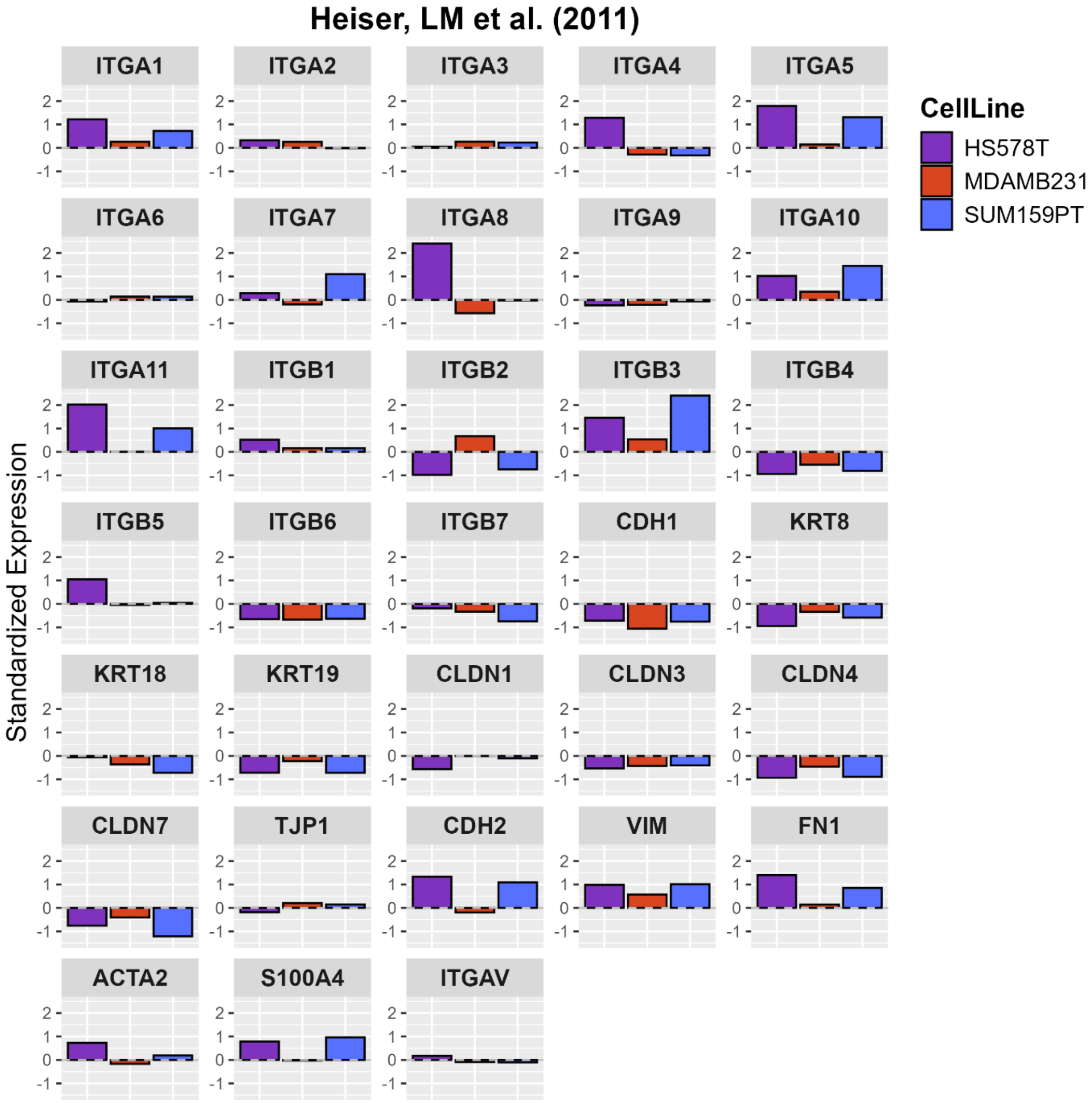

Supplementary Figure 3

Supplementary Figure 3: A) Schematic illustration of the invasion assay setup. Cells were seeded on top of the collagen I matrices and incubated for 7 days. After fixation, nuclei were stained with DAPI and z-stack images were acquired by fluorescence microscopy. An in-house MATLAB script was used to quantify absolute nuclei positions in the z-direction. An exemplary 3D plot of a measured z-stack is shown. B) Schematic illustration of the expansion procedure for non-transmigrated (nt) and transmigrated (t) populations isolated from d→o interface matrices. After 7 days of incubation, cells were enzymatically released using collagenase IV and reseeded into collagen I matrices in T25 flasks for an additional 7 days of expansion prior to RNA isolation.

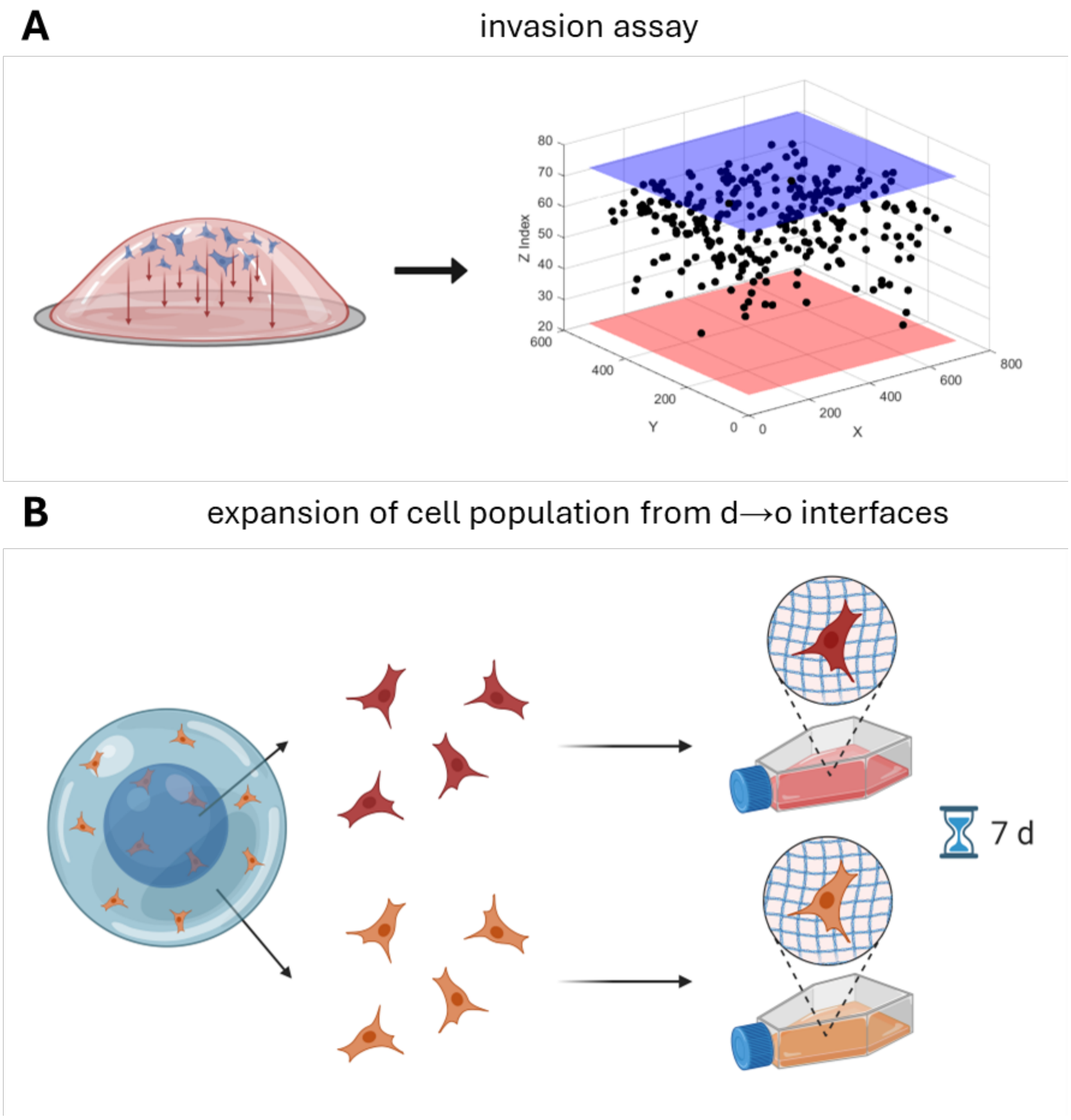

Supplementary Figure 4

Supplementary Figure 4: RNA sequencing analysis of three TNBC cell lines (MDA-MB-231, SUM159PT, Hs578T) cultured in homogeneous dense and open porous collagen I matrices. A) Volcano plots depict DEGs between cells in open porous matrices and dense matrices (adjusted p-value < 0.05, $|\log_2 FC| > 1$). B) PCA illustrating overall transcriptional differences between the three cell lines under these conditions. C) Venn diagram showing the overlap of DEGs across the three cell lines. nt - non-transmigrated, t - transmigrated.

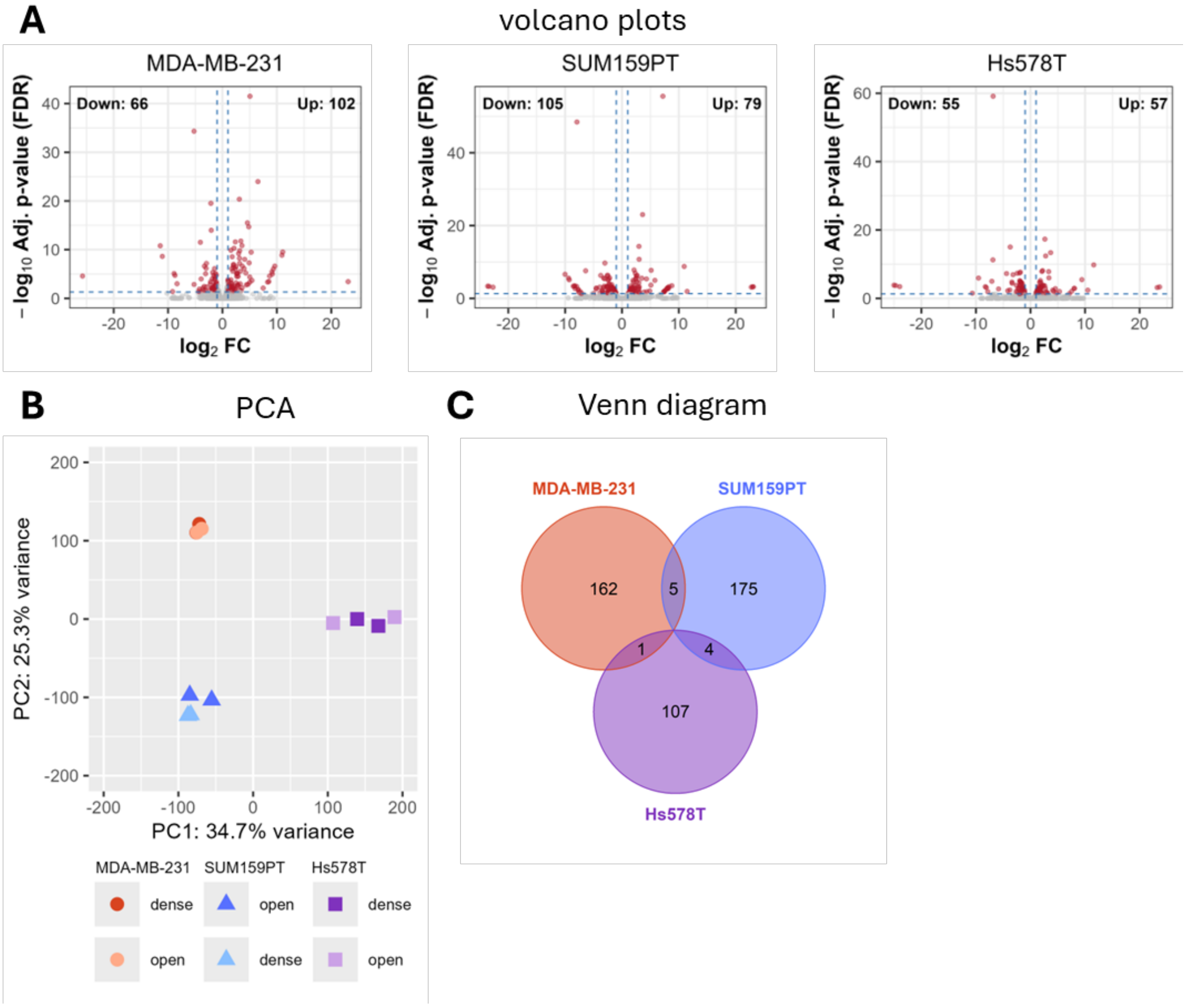

Supplementary Figure 5

Supplementary Figure 5: Reactome pathway classification of the 228 shared DEGs. For the set of commonly regulated genes identified across MDA-MB-231, SUM159PT and Hs578T cells following interface crossing, Reactome pathway enrichment analysis was performed. The plot shows how these genes are distributed across level 2 Reactome pathways within each level 1 ancestor category.

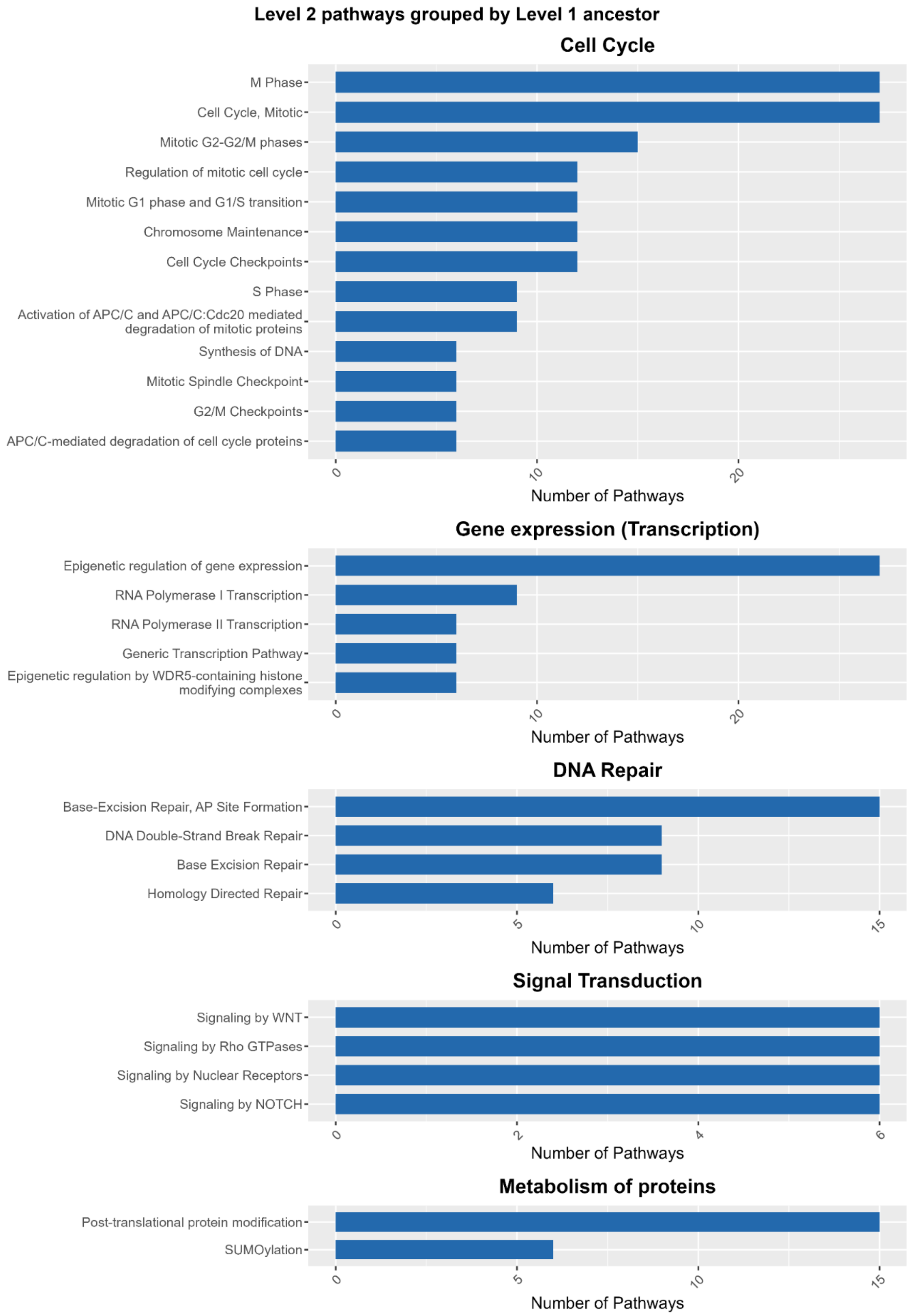

Supplementary Figure 6

Supplementary Figure 6: Single-sample gene set enrichment analysis (ssGSEA) scores for shared pathways shown for individual cell lines MDA-MB-231, SUM159PT and Hs578T. Raw ssGSEA scores were calculated from variance-stabilized RNA-seq expression values using the GSVA package and MSigDB gene sets. Dots represent ssGSEA scores for individual pathways, with paired values between nt and t conditions connected by lines. Horizontal black bars indicate the median ssGSEA score for each condition. d-o – dense-to-open/ d→o, nt – non-transmigrated, t – transmigrated.

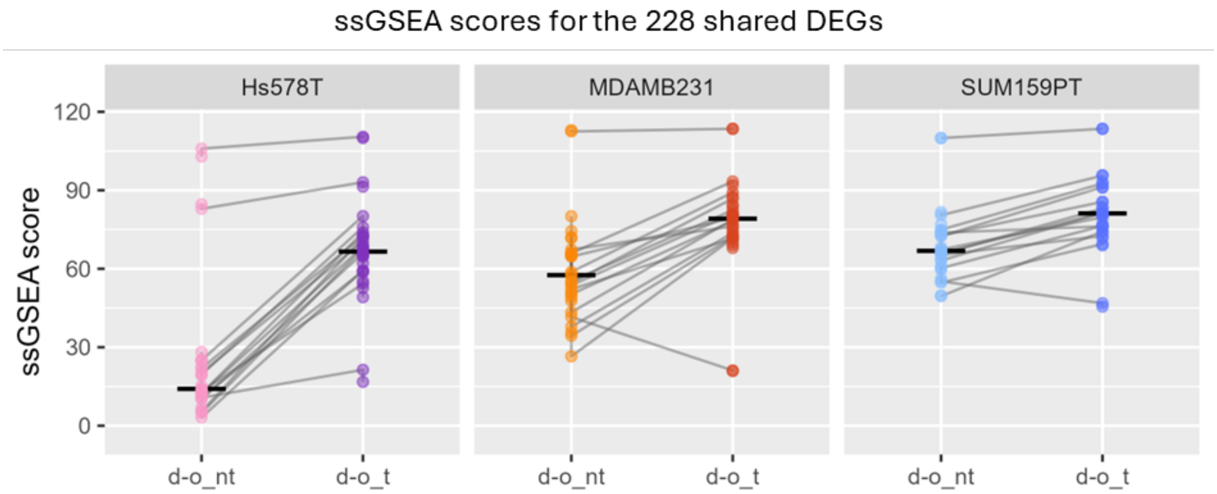

Supplementary Figure 7

Supplementary Figure 7: Clinical association of the shared transmigration-induced gene signature in the METABRIC cohort (Curtis, C et al. (2012) *The genomic and transcriptomic architecture of 2,000 breast tumours reveals novel subgroups*. Nature 486, 346–352). Association of the transmigration-associated gene signature, derived from the 228 shared differentially expressed genes identified across MDA-MB-231, SUM159PT and Hs578T cells, with clinical and pathological features in the METABRIC breast cancer cohort. Signature scores were compared across (A) intrinsic breast cancer subtypes, (B) tumor grade, (C) tumor size, (D) age at diagnosis, (E) number of positive lymph nodes and (F) Nottingham Prognostic Index (NPI). Each dot represents an individual patient sample and red horizontal bars indicate the median signature score per group. Statistical significance was assessed using Wilcoxon Rank-Sum Test.

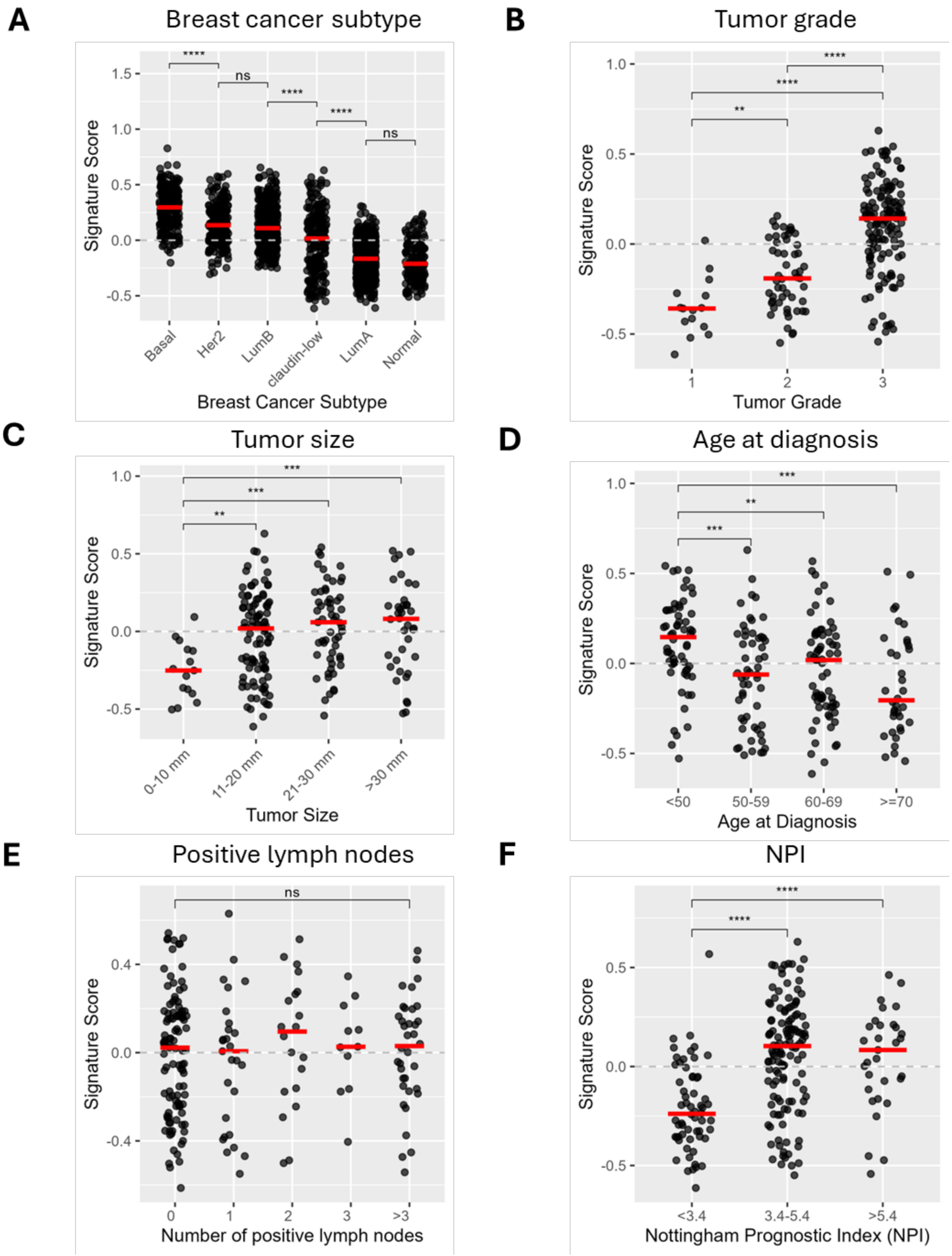

Supplementary Figure 8

Supplementary Figure 8: Overview of Reactome pathway enrichment of all differentially expressed genes for each cell line individually. 3,255 differentially expressed genes (DEGs) in MDA-MB-231, 1,518 in SUM159PT, and 2,622 in Hs578T were analyzed. A) Number of significantly enriched Reactome pathways grouped by Level 1 Reactome pathways for each cell line (Hs578T, MDA-MB-231, and SUM159PT). B) Mean log2 fold change of DEGs associated with each Reactome Level 1 category across cell lines. C) Reactome Level 2 ancestor pathways for selected Level 1 categories (Cell Cycle, DNA Repair, and Extracellular Matrix Organization), displaying the mean log2 fold change of DEGs for each cell line.

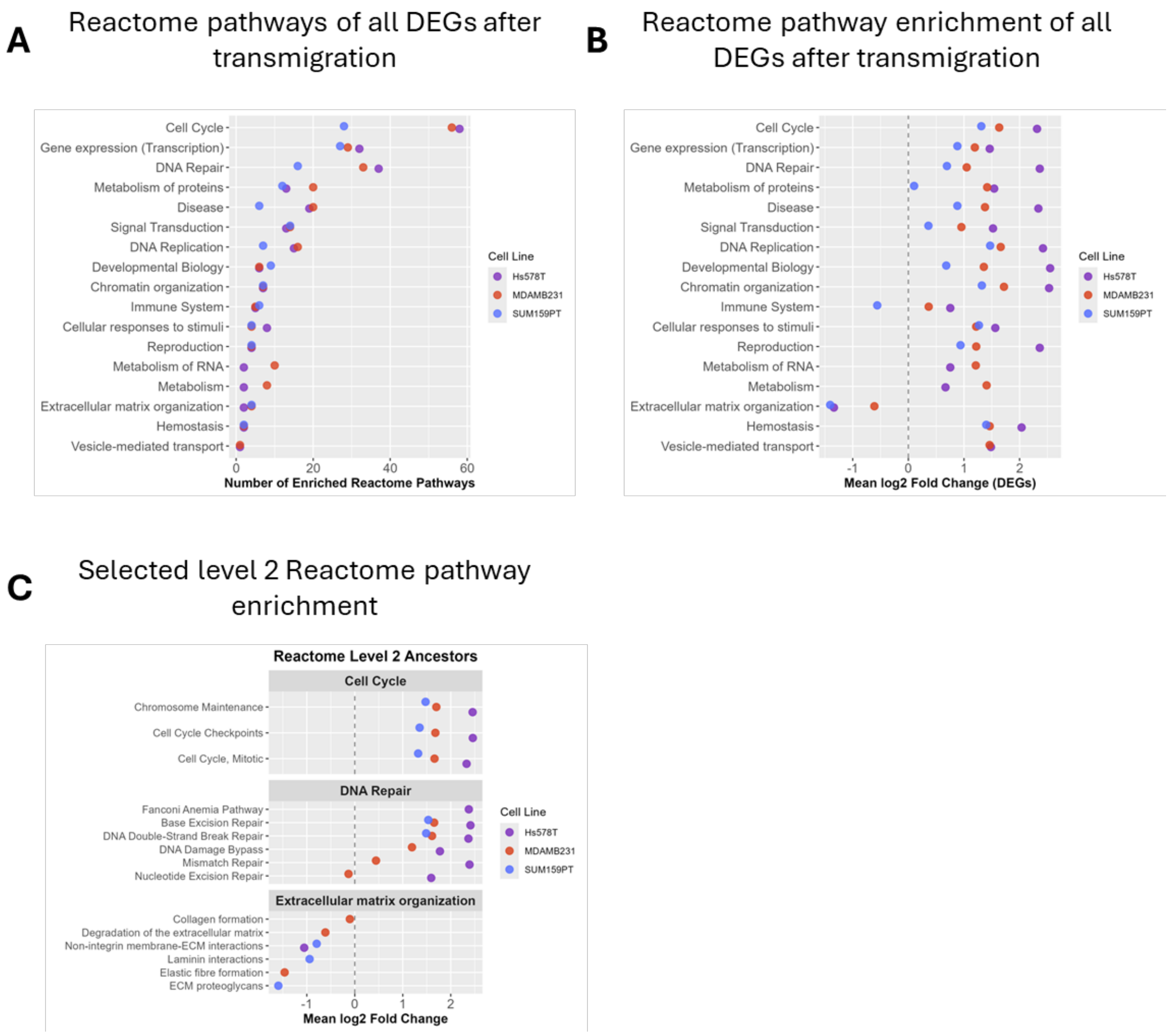

Supplementary Figure 9

Supplementary Figure 9: Log2 fold changes of selected genes from the 228 shared DEGs across Hs578T, MDA-MB-231 and SUM159PT cells following matrix interface crossing. The top panels show representative cell cycle regulators (*CCNB1*, *CCNA2*, *CDC20*, *PLK1*, *MCM4*, *MCM5*, MYBL2) and DNA repair-associated genes (*FANCD2*, *RAD51AP1*, *BLM*). The lower panels show selected EMT markers (CDH1, CDH2), integrin subunits involved in collagen binding (*ITGA1*, *ITGA2*, *ITGA10*, *ITGA11*, *ITGB1*) and the matrix-associated gene *MMP11*.

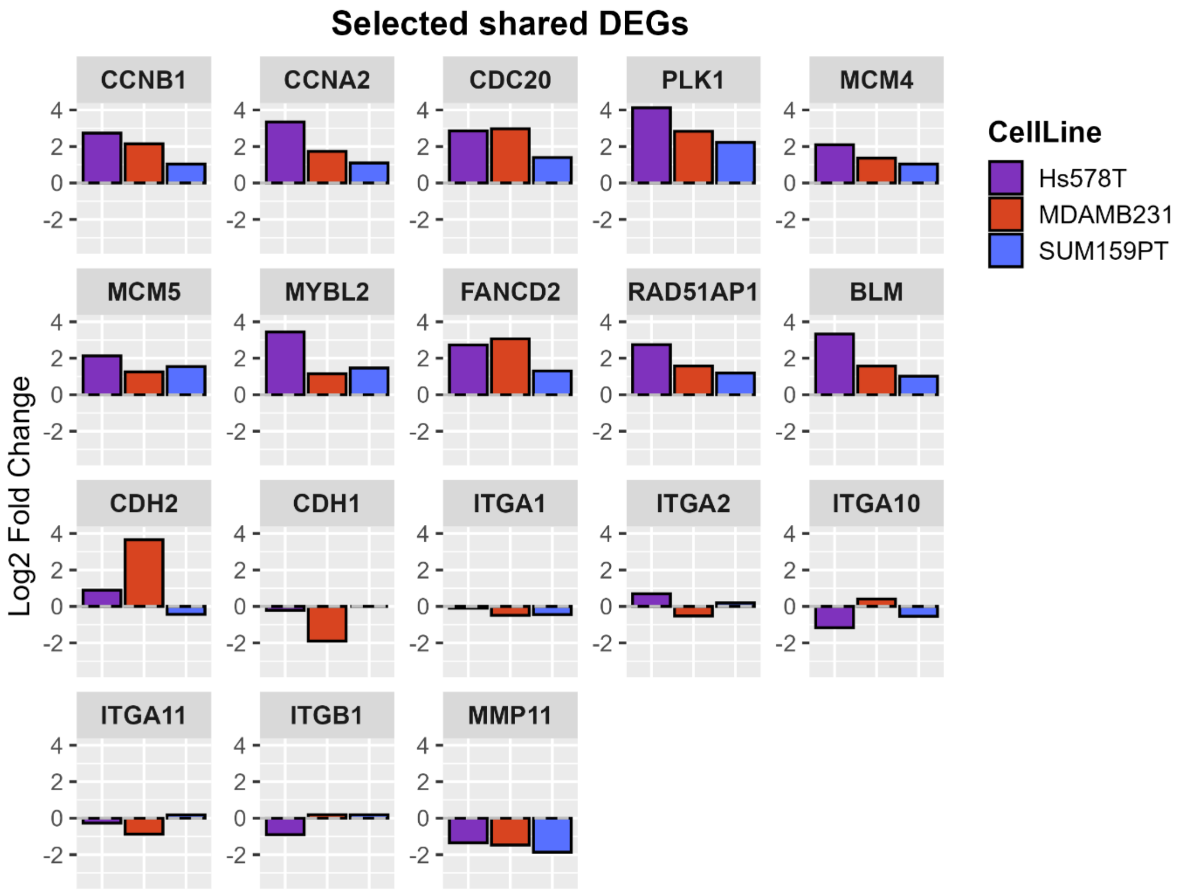

Supplementary Figure 10

Supplementary Figure 10: Percentage of genes within selected Hallmark (Liberzon A et al. (2015) *The Molecular Signatures Database Hallmark Gene Set Collection*. Cell Systems, 1, 6, 417-425) and metastasis-associated gene sets (Wu, S et al. (2025) *metsDB: A Knowledgebase of Cancer Metastasis at Bulk, Single-Cell and Spatial Levels.* Faculty, Staff and Student Publications. 513) covered by differentially expressed genes (DEGs) upon matrix interface crossing. Shown are results for MDA-MB-231, SUM159PT and Hs578T, as well as the subset of 228 shared DEGs conserved across all three cell lines.

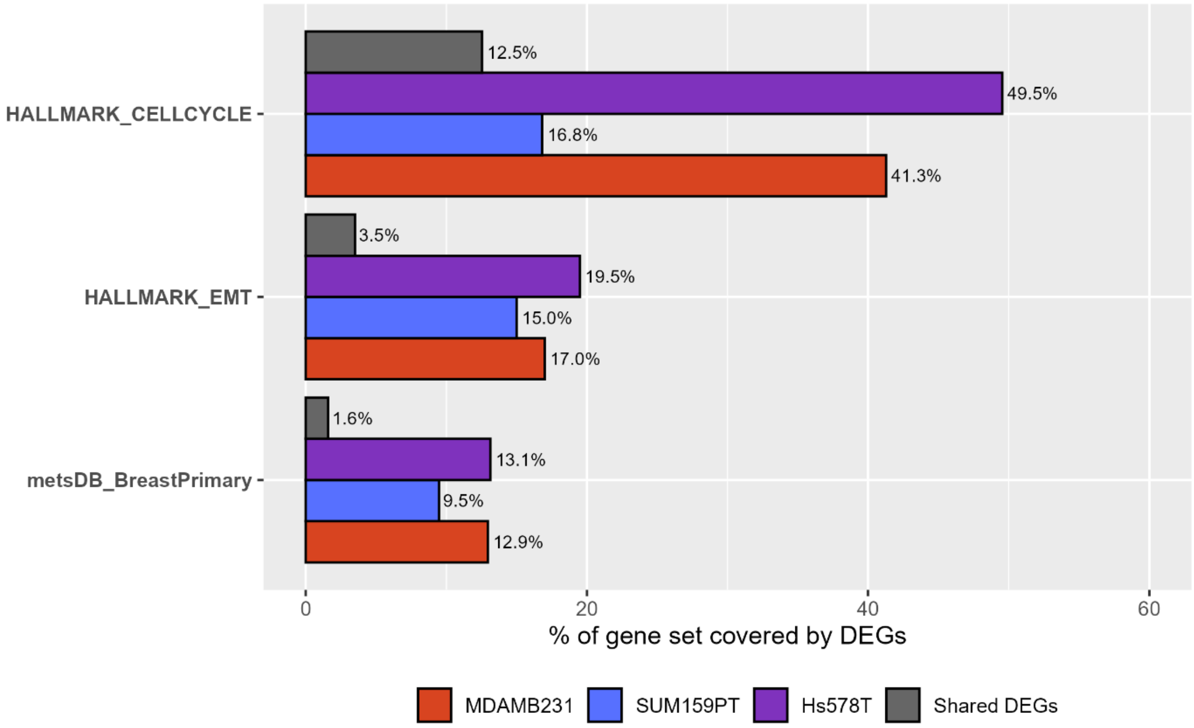

Supplement: Supplementary file 1 — Supporting File: adhm71005‐sup‐0001‐SuppMat.pdf. [file ADHM-15-0-s001.pdf]
